# Identification and Validation of a Prognostic Signature Based on Methylation Profiles and Methylation-Driven Gene *DAB2* as a Prognostic Biomarker in Differentiated Thyroid Carcinoma

**DOI:** 10.1155/2022/1686316

**Published:** 2022-09-17

**Authors:** Gaoda Ju, Lingling Zhang, Wenting Guo, Hao Wang, Xin Zhang, Zhuanzhuan Mu, Yuqing Sun, Di Sun, Han Diao, Sen Miao, Yiran Chen, Tao Xing, Jun Liang, Yansong Lin

**Affiliations:** ^1^Department of Medical Oncology, Key Laboratory of Carcinogenesis & Translational Research (Ministry of Education/Beijing), Peking University Cancer Hospital and Institute, Beijing 100142, China; ^2^Department of Nuclear Medicine, State Key Laboratory of Complex Severe and Rare Diseases, Peking Union Medical College (PUMC) Hospital, Chinese Academy of Medical Sciences & PUMC, Beijing 100730, China; ^3^Beijing Key Laboratory of Molecular Targeted Diagnosis and Therapy in Nuclear Medicine, Beijing 100730, China; ^4^Department of Oncology, Peking University International Hospital, Peking University, Beijing 102206, China; ^5^Department of Oncology, Qingdao Municipal Hospital, School of Medicine, Qingdao University, Qingdao 266011, China; ^6^Department of Pathology, Affiliated Hospital of Jining Medical University, Jining 272000, China

## Abstract

Recurrence is the major death cause of differentiated thyroid carcinoma (DTC), and a better understanding of recurrence risk at early stage may lead to make the optimal medical decision to improve patients' prognosis. The 2015 American Thyroid Association (ATA) risk stratification system primary based on clinic-pathologic features is the most commonly used to describe the initial risk of persistent/recurrent disease. Besides, multiple prognostics models based on multigenes expression profiles have been developed to predict the recurrence risk of DTC patients. Recent evidences indicated that aberrant DNA methylation is involved in the initiation and progression of DTC and can be useful biomarkers for clinical diagnosis and prognosis prediction of DTC. Therefore, there is a need for integrating gene methylation feature to assess the recurrence risk of DTC. Gene methylation profile from The Cancer Genome Atlas (TCGA) was used to construct a recurrence risk model of DTC by successively performed univariate Cox regression, LASSO regression, and multivariate Cox regression. Two Gene Expression Omnibus (GEO) methylation cohorts of DTC were utilized to validate the predictive value of the methylation profiles model as external cohort by receiver operating characteristic (ROC) curve and survival analysis. Besides, CCK-8, colony-formation assay, transwell, and scratch-wound assay were used to investigate the biological significance of critical gene in the model. In our study, we constructed and validated a prognostic signature based on methylation profiles of *SPTA1, APCS,* and *DAB2* and constructed a nomogram based on the methylation-related model, age, and AJCC_T stage that could provide evidence for the long-term treatment and management of DTC patients. Besides, in vitro experiments showed that *DAB2* inhibited proliferation, colony-formation, and migration of BCPAP cells and the gene set enrichment analysis and immune infiltration analysis showed that *DAB2* may promote antitumor immunity in DTC. In conclusion, promoter hypermethylation and loss expression of *DAB2* in DTC may be a biomarker of unfavorable prognosis and poor response to immune therapy.

## 1. Introduction

Thyroid cancer has been the fifth most common cancer expected to be diagnosed in female and is the most common endocrine cancer according to the global cancer statistics 2020 [1]. Differentiated thyroid carcinoma (DTC) is the most common thyroid cancer, accounting for more than 90% of cases [1]. Although the long-term prognosis is favorable in the majority of patients with DTC, especially in the most popular subtype of papillary thyroid cancer (PTC), up to 30% patients experience recurrent disease after initial therapy [2, 3]. Accurate assessment of the recurrence risk would be conducive to the clinical decision making, so as to reduce the recurrence rate and improve the quality of life for DTC patients [4]. The 2015 American Thyroid Association (ATA) risk stratification system, mainly based on the pathological characteristics such as tumor size, lymph node metastasis, and genetic feature such as BRAFV600E mutation, is the most commonly used to describe the initial risk of persistent/recurrent disease [5]. The response to therapy further integrates the real-time biochemical and imaging results, which is helpful for the dynamic monitoring of recurrence. Of note, over the recent decades, the transcriptional profiles and clinical prognosis information of tumor samples integrated by The Cancer Genome Atlas (TCGA) are beneficial for researchers to construct gene signatures for diagnosis and predicting the prognosis of patients with cancer [6–11]. However, these currently used gene signatures that solely dependent on data from TCGA cohort are not integrated to the risk stratification system and still limited to predict the recurrence risk early and accurately of patients with PTC.

DNA methylation is involved in transcriptional regulation and genome stability [12], and aberrant DNA methylation can lead to the development of many types of cancer [13, 14], also including PTC [15]. Moreover, DNA methylation signatures can be used as biomarkers for clinical diagnosis and prognosis prediction of cancer [16]. For instance, Langdon et al. identified a panel of DNA methylation biomarkers for early diagnosis of renal cell carcinoma. Besides, DNA methylation of *SPEG* located at Chr2:220325443-220326041 could potentially regulate oropharyngeal cancer survival [17]. Shen et al. reported that hypermethylation of SSTR2 promoter could be used to predict prognosis of laryngeal squamous cell carcinoma in males [18]. The methylation profiles of *GSTP1*, *HIC1*, and *RASSF1A* were associated with the recurrence of bladder cancer [19]. Accumulating studies have reported that aberrant DNA methylation can be useful biomarkers for clinical diagnosis of DTC. For example, Wei et al. reported that hypermethylation of both PTEN and DAPK is capable of discriminating malignant thyroid tissues from benign lesions [20]. Besides, a panel of 3 DNA methylation biomarkers discriminated thyroid cancers from benign thyroid lesions was identified by Park et al. [21]. Several studies have reported aberrant DNA methylation of tumor suppressor genes was associated with tumor aggressiveness in PTC [22, 23]. However, a panel of gene methylation profiles for predicting the recurrence of DTC is still lacking. Hence, developing and validating a signature based on methylation profiles is of important clinical significance.

In our study, we established and validated a panel of methylation profiles of *SPTA1*, *APCS*, and *DAB2* for predicting the recurrence risk of DTC patients with data from TCGA. The significant effectiveness of our methylation-related panel in predicting recurrence of DTC patients was validated by GSE51090 and GSE97466 cohorts. According to previous literatures [24], promoter hypermethylation of *DAB2* associated with the loss expression of *DAB2* in many types of human cancers, such as nasopharyngeal carcinoma [25], breast cancer [26], and esophageal squamous cell carcinomas [27].

Our study first reported that *DAB2* was hypermethylation and low expressed in DTC samples and associated with proliferation, survival, and migration of DTC cells.

## 2. Materials and Methods

### 2.1. Data Acquisition

The methylation profiles, transcriptional profiles, and clinical information of TCGA-THCA were downloaded from TCGA website (https://portal.gdc.cancer.gov/). The data of 58 normal thyroid tissues and 497 PTC patients with survival information were extracted for our study (Table S1). Two external methylation cohorts of DTC were downloaded from Gene Expression Omnibus (GEO) website (https://www.ncbi.nlm.nih.gov/geo/). The survival information of the 2 GEO cohorts (GSE51090, GSE 97466) was extracted from previous articles (Tables S2 and S3) [28, 29]. For methylation profiles of genes, mean values were taken for multiple probes with an identical gene symbol. DTC patients with local recurrence or distant metastasis or biochemical evidence of disease were considered as recurrence DTC patients. Besides, GSE51090 cohorts and GSE97466 cohorts were integrated as an independent GEO cohort for validating the results. ComBat function was utilized to remove the batch effect between GSE51090 cohorts and GSE97466 cohorts (Figure S1).

### 2.2. Identified Differential Methylation Genes (DMGs) Between DTC and Normal Thyroid Tissues

According to the previous studies [30, 31], the R package “limma” [32] was used to identify DMGs with |log2 fold change| > = 0.2, |*β*tumor − *β*normal| > 0.1, and false discovery rate (FDR) < 0.05.

### 2.3. Constructed and Validated a Model Based on Methylation Profiles of 3 DMGs

The TCGA cohort was utilized to construct a DMGs methylation profiles-related recurrence risk model of DTC patients. Univariate Cox regression, lasso regression, and stepwise multivariate Cox regression were successively performed to identify DMGs closely related to the recurrence of PTC with “survminer,” “survival,” and “glmnet” package [33]. To validate the prognostic prediction value of the model, receiver operating characteristic (ROC) curve analysis and survival analysis were performed by R software with “survivalROC” package [34], and data form both TCGA and GEO cohorts. The riskScore_me of 497 PTC patients were calculated as follows:

(1) $$\begin{array}{c} \text{riskScor}{\text{e}}_{\text{me}}=\overset{n}{\underset{i=1}{\sum }}coef\left(i\right)beta\text{ value}\left(i\right). \end{array}$$

X-Title software was utilized to find the best cutoff riskScore_me which discriminate PTC patients with High Score and Low Score.

### 2.4. Tumor Infiltrating Cells Analysis

ESTIMATE [35] and xCell [36] (https://xcell.ucsf.edu/) analysis were performed by R software according to the instruction in the website.

### 2.5. Public Online Database Analysis

The Gene Expression Profiling Interactive Analysis (GEPIA) [37] database (http://gepia.cancer-pku.cn/) was used to plot the mRNA expression of *DAB2*, *SPTA1*, and *APCS* between PTC samples and normal thyroid tissues with data from TCGA and GTEx. Metascape [38] database (https://metascape.org) was used to perform Kyoto Encyclopedia of Genes and Genomes (KEGG) analysis.

### 2.6. Constructed and Validated a Nomogram Based on Methylation Profiles and Clinical Parameters

RiskScore_me and clinical parameters (AJCC_T, AJCC_N, AJCC_M, stage, age, histological_type, and sex) of 497 PTC patients were enrolled for further analysis. Univariate and stepwise multivariate Cox regression analysis were performed to construct a recurrence risk model combined riskScore_me, age, and AJCC_T. To validate the prognostic prediction value of the model, ROC curve analysis, predict function, calibration curve, and survival analysis were performed by R software. Finally, a nomogram based on methylation profiles, age, and AJCC_T was established for predicting recurrence probability of DTC patients by R software with “rms” [39] and “foreign” package. Decision curve analysis (DCA) was performed to compare the predicting value of our model with other 2 previous models by R software with “ggDCA” package [40].

### 2.7. GSEA Analysis

GSEA (Gene Set Enrichment Analysis) [41, 42] was performed by R software with “clusterProfiler” package. The KEGG pathways with *P* < 0.05 and FDR < 0.05 were considered as pathways that are significantly correlated with expression of *DAB2* in PTC samples.

### 2.8. Human Thyroid Carcinoma Specimens

34 thyroid carcinoma specimens along with paired adjacent normal thyroid specimens collected from Affiliated Hospital of Jining Medical University was approved by the Ethics Committee of Affiliated Hospital of Jining Medical University. The approval number was 2021-08-C015. All participants provided written informed consent.

### 2.9. Immunohistochemistry

Immunohistochemistry (IHC) staining for *DAB2* (Proteintech, 10109-2-AP, 1 : 500) was performed by standard protocol as described before [43]. IHC score of *DAB2* was determined by multiplying the score of staining intensity with the score of frequency of positive staining cells. Staining intensity was defined as negative (0), weak (1), moderate (2), and strong (3). Frequency of positive cells was defined as less than 5% (0), 5%-25% (1), 26%-50% (2), 51%-75% (3), and more than 75% (4).

### 2.10. Cell Lines and Culture Conditions

Human thyroid cancer cell line BCPAP was acquired from ATCC and cultivated in DMEM medium (Gibco) with 10% fetal bovine serum (FBS) (Gibco) and 1% penicillin-streptomycin (Gibco), at 37°C with 5% CO2.

### 2.11. Plasmids and Lentivirus Production

*DAB2* coding sequence was cloned into the viral skeleton plasmid PCDH-3 flag-tagged vector for stable expression in BCPAP cells.

### 2.12. Western Blotting

Cell proteins were extracted by denatured buffer and then quantified by pierce BCA protein assay (Thermo Scientific). The protein lysate was separated on SDS-PAGE, transferred to NC membranes (Millipore), blocked, and then detected by primary antibody *DAB2* (Proteintech, 10109-2-AP, 1 : 2000) or *β*-Actin (Proteintech 20536-1-AP,1 : 2000) and HRP-conjugated secondary antibody (Sigma), followed by being exposed with enhanced chemiluminescence (ECL).

### 2.13. Cell Growth Assay

Lentivirus infected stable BCPAP cells were seeded into 96-well plates and cultured in 10% FBS DMEM (2000 cells per well, five parallel wells). Then, the cells were collected at different points in time, and cell number in each well was counted by CCK-8 reagent. The absorbance at 450 nm of each well was measured to calculate the cell proliferation.

### 2.14. Colony-Forming Assay

Lentivirus infected stable BCPAP cells were seeded into 6-well plate at a density of 200 cells per well and cultured in DMEM with 10% FBS for 3 weeks. Then, colonies per well were stained by 0.1% crystal violet and counted by ImageJ software.

### 2.15. Transwell Assay

Lentivirus infected stable cells were seeded in the upper chamber of transwell chamber (24-well, 8 *μ*m pore, Corning) in 200 *μ*L of serum-free DMEM (1 × 10^5^ cells per well, 3 parallel wells) and 800 *μ*L of 10% FBS DMEM was added to the lower chamber and incubated for 36 h at 37°C. After removing the cells at the upper surface of the membrane, the cells passed through the filter were successively fixed with 4% paraformaldehyde, stained with 0.1% crystal violet solution, and photographed by inverted fluorescence microscope.

### 2.16. Scratch-Wound Assay

BAPCP cells were seeded in 6-well plates and then incubated for 24 h to reach approximately 80% confluence. The cell monolayer was scratched using a sterile 100 *μ*L pipette tip. Then, the cells were treated with serum-free medium. The wounds were photographed at 0 and 36 h, and migration distance of the cell was calculated by ImageJ (migratory ratio: 0-36 h width of wound/0 h width of wound).

### 2.17. Statistical Analysis

Two-tailed *t* test was utilized to analyze the difference between two groups. Log-rank test was utilized for survival analysis.

## 3. Results

### 3.1. Constructed and Validated a Recurrence Risk Model of DTC Based on Methylation Profiles of 3 DMGs

**Figure 1:**
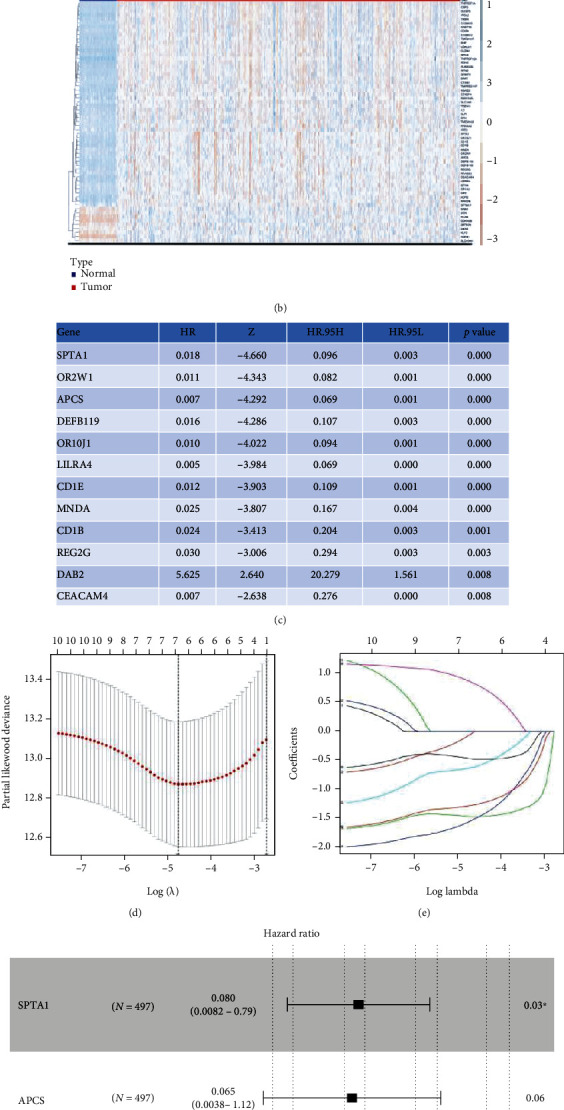
Constructed a recurrence risk model based on methylation profiles of 3 genes. (a) Venn diagrams of intersection genes. (b) The heat map of DMGs' methylation profiles between DTC and normal thyroid samples. (c) DMGs which had significant value for predicting prognosis of DTC patients identified by the univariate Cox regression analysis. (d) LASSO coefficient-Log (*λ*) curve of 12 DMGs. (e) Selecting the optimal log (*λ*) in LASSO model. (f) A recurrence risk model based on methylation profiles of 3 genes. ^∗^*P* < 0.05. DMGs: differentially methylation genes.

**Figure 2:**
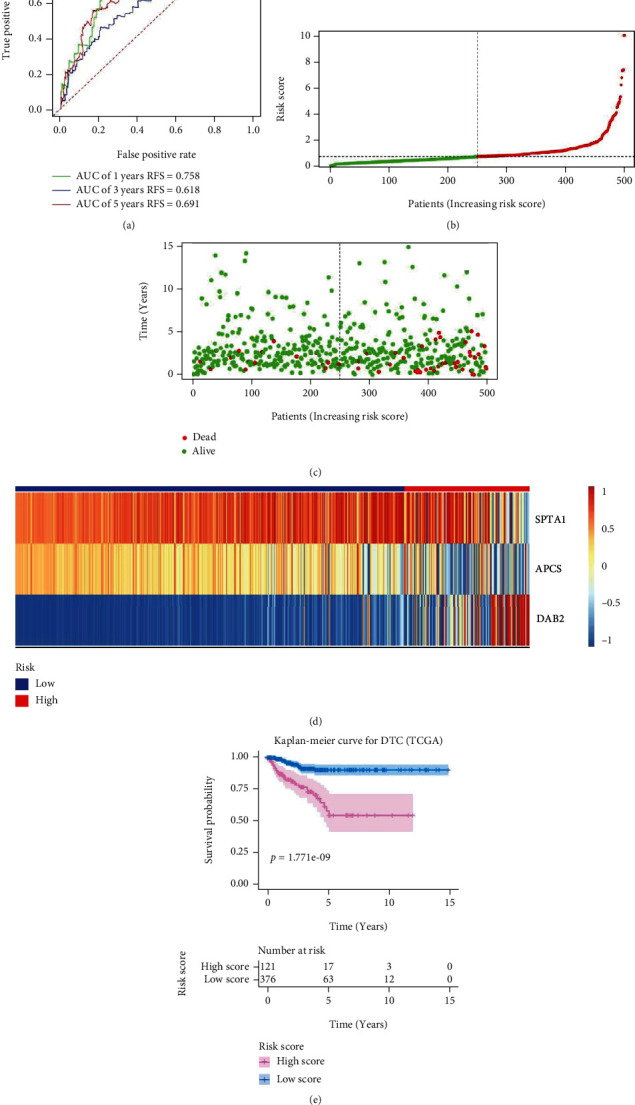
Validated the recurrence risk model based on methylation profiles of 3 genes in the internal cohort. (a) Time-dependent receiver operating characteristic curves for predicting 1-year, 3-year, and 5-year recurrence free survival in the internal cohort. (b) Risk score distribution of DTC patients in the Train cohort. (c) Survival status of DTC patients in the internal cohort. (d) Methylation profiles of 3 genes in high risk group and low risk group by heat map analysis. (e) Kaplan-Meier survival curves for recurrence free survival. AUC: area under the curve.

**Figure 3:**
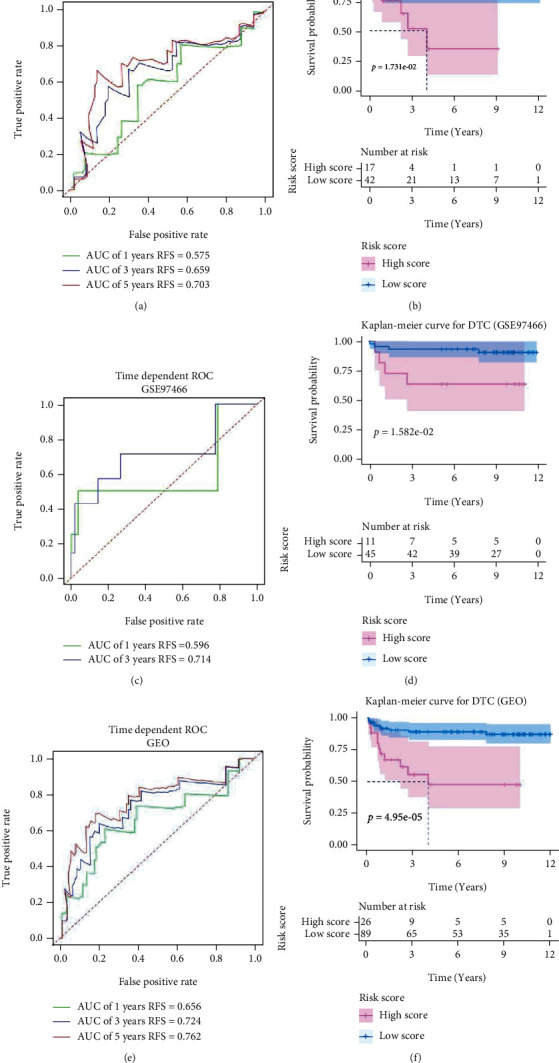
Validated the recurrence risk model based on methylation profiles of 3 genes in the external cohort. (a) Time-dependent receiver operating characteristic curves for predicting 1-year, 3-year, and 5-year recurrence free survival in the GSE51090 cohort. (b) Kaplan-Meier survival curves for recurrence free survival in the GSE51090 cohort. (c) Time-dependent receiver operating characteristic curves for predicting 1-year and 3-year recurrence free survival in the GSE97466 cohort. (d) Kaplan-Meier survival curves for recurrence free survival in the GSE97466 cohort. (e) Time-dependent receiver operating characteristic curves for predicting 1-year, 3-year, and 5-year recurrence free survival in the GEO cohort (GSE51090 combined with GSE97466). (f) Kaplan-Meier survival curves for recurrence free survival in the GEO cohort. AUC: area under the curve.

The methylation profiles of 10362 genes in the intersection of TCGA and 2 GEO cohorts were extracted for our research (Figure 1(a)). 61 DMGs were identified between DTC and normal thyroid samples (Figure 1(b)). By performing univariate Cox regression with data from TCGA cohort, DMGs with *P* < 0.01 were selected for further analysis (Figure 1(c)). Then by successively performing lasso regression (Figures 1(d) and 1(e)) and stepwise multivariate regression analysis, methylation profiles of *SPTA1*, *APCS*, and *DAB2* were utilized to construct a recurrence risk mode of PTC patients (Figure 1(f)). In the TCGA cohort, the area under the ROC curve (AUC) of 1-year, 3-year, and 5-year recurrence free survival were 0.758, 0.618, and 0.691, respectively (Figure 2(a)). The riskScore distribution, survival status of PTC patients, and the methylation profiles heat map of 3 genes between Low Score and High Score PTC samples are shown in Figures 2(b)–2(d). Kaplan-Meier result indicated that PTC patients in Low Score group had longer recurrence free survival (RFS) than those in High Score group (Figure 2(e)). As external validated cohorts, the AUC of 1-year, 3-year, and 5-year recurrence free survival were 0.575, 0.659, and 0.703 in the GES51090 cohort, respectively (Figure 3(a)) and PTC patients in High Score group had worse RFS than those in Low Score group (Figure 3(b)). Similarity, the AUC of 1-year and 3-year recurrence free survival were 0.596 and 0.714 (Figure 3(c)) and High Score PTC patients had worse RFS (Figure 3(d)) in the GES97466 cohort. Finally, the data of 2 GEO cohorts were integrated as a single external validated GEO cohort for further analysis. The batch effects between GSE51090 and GSE97466 were removed by “combat” function of R “sva” package (Figure S1). The result showed that the AUC of 1-year, 3-year, and 5-year recurrence free survival were 0.656, 0.724, and 0.762 (Figure 3(e)) and High Score PTC patients had shorter RFS (Figure 3(f)) in the single GEO cohort. Obviously, the results of validation were consistent between internal and external cohorts.

### 3.2. Constructed a Nomogram of DTC Based on Methylation Profiles of 3 Genes, Age, and AJCC_T

**Figure 4:**
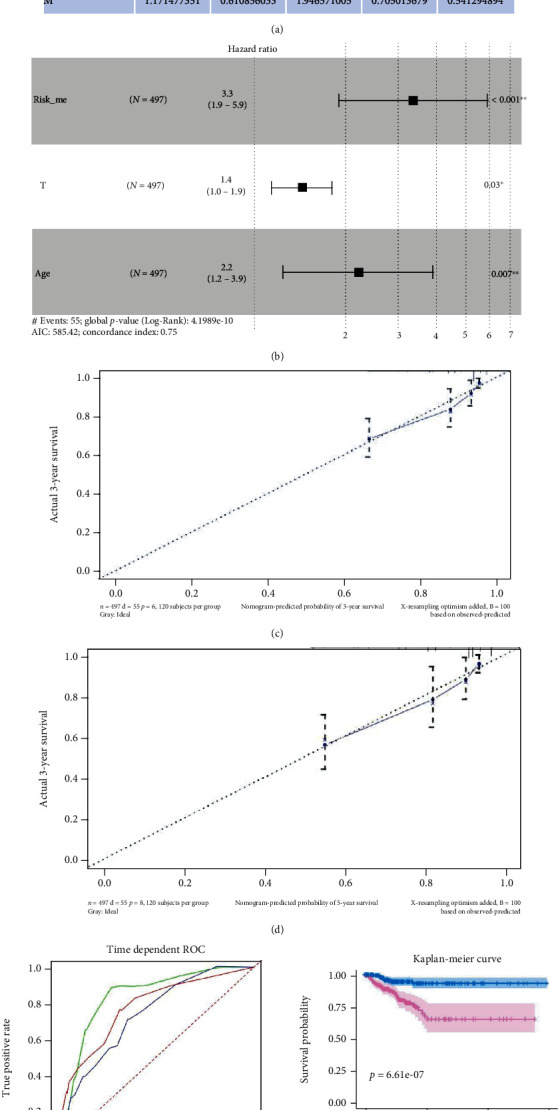
Constructed and validated a recurrence risk model based on riskScore_me and clinical parameters. (a) Clinical factors which had significant value for predicting prognosis of DTC patients identified by univariate Cox regression analysis. (b) riskScore_me, AJCC_T, and age was utilized to construct recurrence risk model of DTC patients. (c) Calibration curve of 3-year RFS of recurrence risk model based on risk_me and clinical parameters. (d) Calibration curve of 5-year RFS of recurrence risk model based on risk_me, risk_immu, and clinical parameters. (e) Time-dependent receiver operating characteristic curves for predicting 1-year, 3-year, and 5-year recurrence free survival. (f) Kaplan-Meier survival curves for recurrence free survival. AUC: area under the curve.

**Figure 5:**
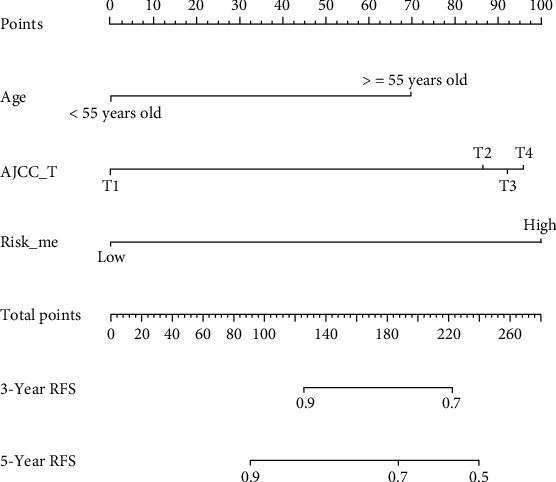
A nomogram was developed based on riskScore_me, AJCC_T, and age.

By performing univariate Cox (Figure 4(a)), 3 clinical factors (age, AJCC_T, and stage) (*P* < 0.05) were selected to experience stepwise multivariate Cox regression analysis with riskScore_me and a recurrence risk model of DTC combing age, AJCC_T, and risk_me was constructed (Figure 4(b)). The calibration curve for 3-year and 5-year recurrence free survival shown a consistency between predicted value of model and actual observed value (Figures 4(c) and 4(d)). The AUC of 1-year, 3-year, and 5-year RFS were remarkably increased to 0.838, 0.734, and 0.775, respectively (Figure 4(e)). The Kaplan-Meier analysis indicated that the DTC patients in Low Score group had significantly longer RFS than those in High Score group (Figure 4(f)). Finally, we constructed a nomogram based on methylation-related recurrence risk model, age, and AJCC_T for predicting RFS probability of DTC patients (Figure 5).

### 3.3. Compared the Prognosis-Predicting and Clinical Value of Our Model with 2 Previous Recurrence Risk Models

**Figure 6:**
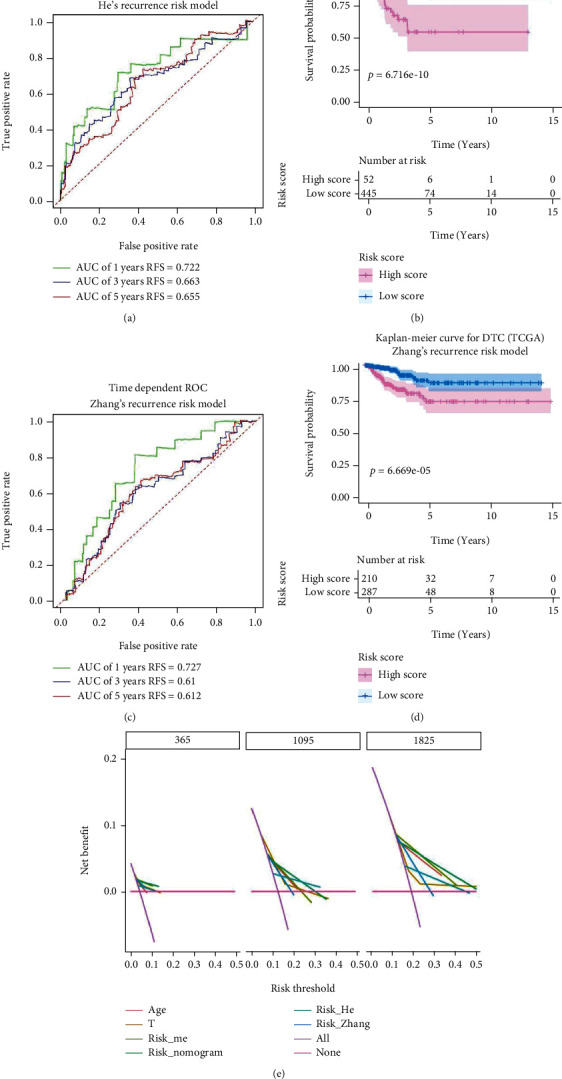
Compared the prognosis-predicting and clinical value of our models with 2 previous recurrence risk models. (a) Time-dependent receiver operating characteristic curves for predicting year, 3-year and 5-year recurrence free survival with He et al.'s recurrence risk model. (b) Kaplan-Meier survival curves for recurrence free survival based on He et al.'s recurrence risk model. (c) Time-dependent receiver operating characteristic curves for predicting 1-year, 3-year, and 5-year recurrence free survival with Zhang et al.'s recurrence risk model. (d) Kaplan-Meier survival curves for recurrence free survival based on Zhang et al.'s recurrence risk model. (e) Decision curve analysis.

Two previous recurrence risk models were reconstructed and revalidated with our data from TCGA-THCA according to the riskScore-calculating equations from the 2 previous articles. By ROC and survival analysis, we found that both He et al.'s [44] and Zhang et al.'s [8] recurrence risk model had good performance in predicting RFS of DTC patients (Figures 6(a)–6(d)). However, by performing DCA, we found that the model based on methylation profiles had outstanding performance in predicting 5-year RFS of patients with DTC. Of note, the nomogram which combined risk_me, age, and AJCC_T would dramatically improve the long-term treatment and management of DTC patients (Figure 6(e)).

### 3.4. *DAB2* Was Abnormally Expressed Between DTC Samples and Normal Thyroid Tissues

**Figure 7:**
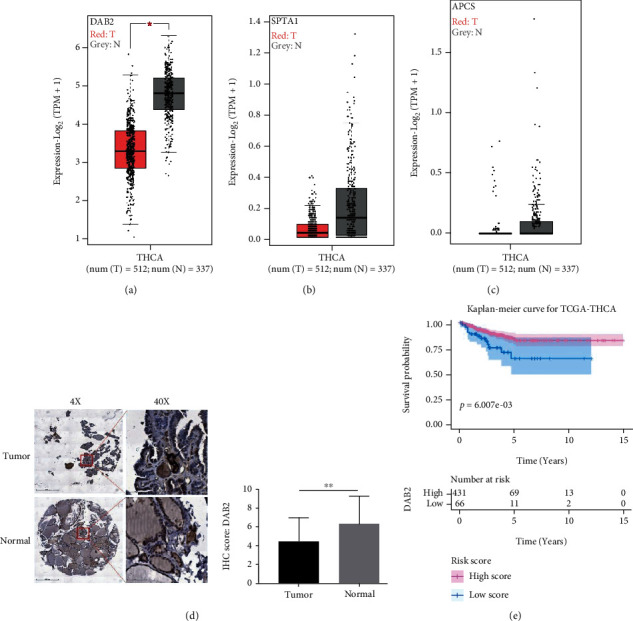
*DAB2* was abnormally expressed in DTC samples. (a) mRNA expression of *DAB2* between DTC and normal thyroid tissues. (b) mRNA expression of *SPTA1* between DTC and normal thyroid tissues. (c) mRNA expression of *APCS* between DTC and normal thyroid tissues. (d) Protein expression of *DAB2* between DTC and normal thyroid tissues. (e) Kaplan-Meier survival curves for recurrence free survival based on the mRNA expression of *DAB2*. T: thyroid cancer tissues; N: normal tissues. ^∗^*P* < 0.05, ^∗∗^*P* < 0.01.

For better understanding the biological significance of genes in the model, GEPIA was utilized to investigate the differences in mRNA expression between DTC and normal thyroid tissues. Boxplot showed that the mRNA expression of *DAB2* was downregulated in DTC samples compare to normal thyroid tissue (*P* < 0.05) (Figure 7(a)) while the mRNA expressions of *SPTA1* and *APCS* (Figures 7(b) and 7(c)) were no significant difference between cancer and para-cancer tissues. Then, we found that *DAB2* expression was lower in DTC tissues than that in normal thyroid tissues at the protein level by IHC staining for *DAB2* in our specimens (Figure 7(d)) and found that DTC patients with high expression of *DAB2* had a longer RFS compare to that with low expression *DAB2* (Figure 7(e)).

Overall, *DAB2* associated with pathological feature and prognosis of DTC patients.

### 3.5. *DAB2* Overexpression Inhibited Proliferation, Colony-Formation and Migration of BCPAP Cells

**Figure 8:**
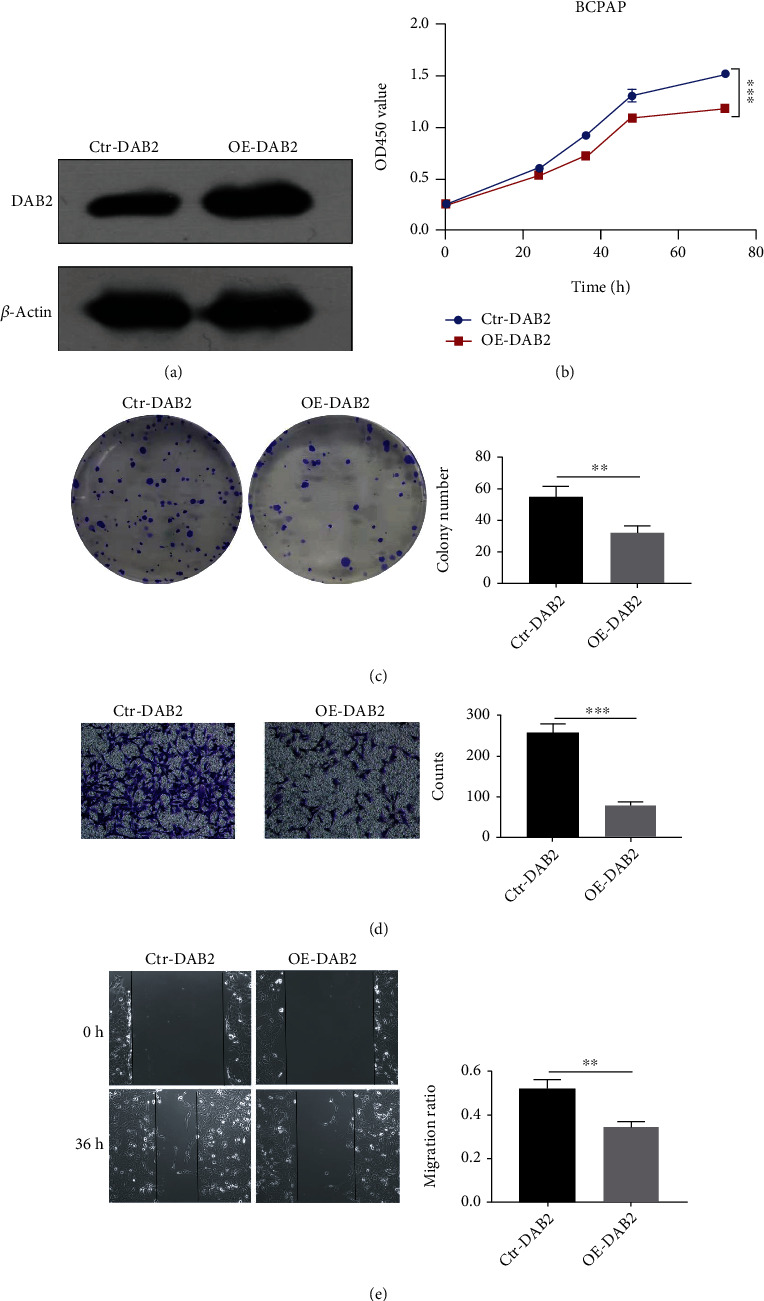
Overexpression of *DAB2* inhibited proliferation, colony-formation, and migration of BCPAP cells. (a) The protein level of *DAB2* and *β*-Actin in BCPAP cells after transfection of vector for *DAB2* stable expression (OE-*DAB2*) or control vector (Ctr-*DAB2*) were determined by western blot assay. (b) CCK8 assay. (c) Colony-formation assay. (d) Transwell assay. (e) Scratch-wound assay. ^∗∗^*P* < 0.01, ^∗∗∗^*P* < 0.001.

To explore whether does *DAB2* impacts DTC cells, we constructed and validated a *DAB2* stable expressed BCPAP cells in our study (Figure 8(a) and Figure S2). In the results from CCK8 assay, colony-formation assay demonstrated that overexpression of *DAB2* inhibited BCPAP cells proliferation (Figure 8(b)) and colony-formation (Figure 8(c)). Besides, transwell assay (Figure 8(d)) and scratch-wound assay (Figure 8(e)) showed that *DAB2* inhibited the migratory ability of BCPAP cells. For better understanding the potential biological significance of *DAB2* in the progress of DTC, all 497 PTC patients were assigned into High *DAB2* and Low *DAB2* group based on the median mRNA expression of *DAB2* using data from TCGA. The differential expression genes (DEGs) with FDR < 0.05 and |log2 fold change| > = 1 between High *DAB2* and Low *DAB2* group were identified with “limma” package (Figure S3A), and the result of KEGG analysis using these DEGs as input showed that *DAB2* regulated the migration and proliferation of DTC may through regulating signaling pathways such as Cell adhesion molecules, NF-kappa B signaling pathway, ECM-receptor interaction, and PI3K-Akt signaling pathway (Figure S3B).

### 3.6. *DAB2* may Promote Antitumor Immunity in PTC

**Figure 9:**
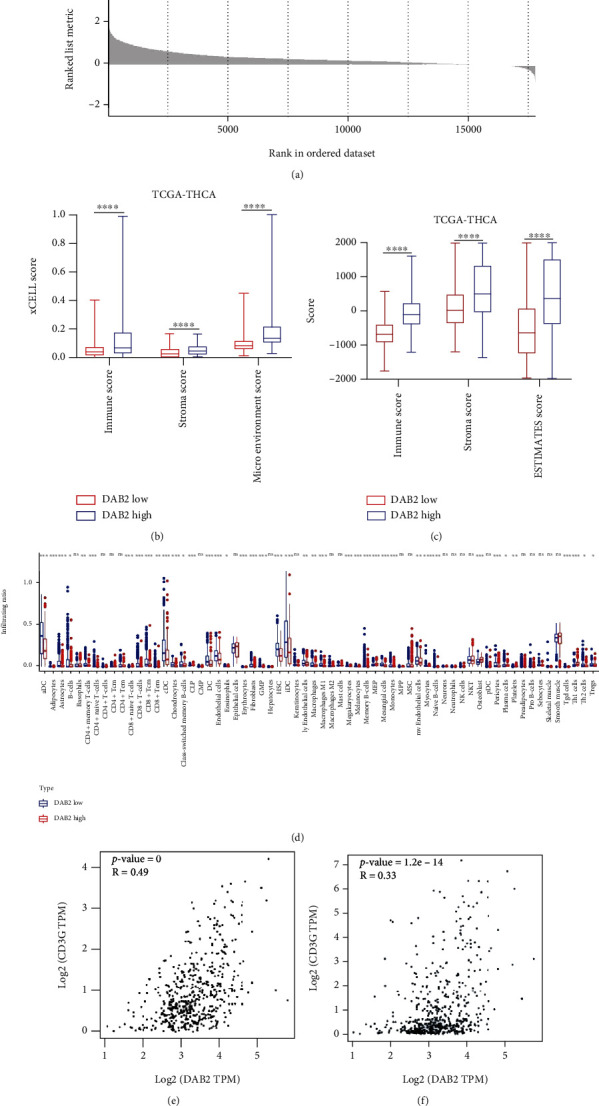
*DAB2* expression associated with immune infiltration in DTC. (a) GSEA plot. (b) DTC patients with high *DAB2* expression had higher level of immune score by xCell than that with low *DAB2* expression. (c) DTC patients with high *DAB2* expression had higher level of immune score by ESTIMATE than that with low *DAB2* expression. (d) Immune cell types infiltration rates between DTC samples with high *DAB2* expression and DTC samples with low *DAB2* expression. (e) *DAB2* expression was positively correlated with CD3G expression in DTC samples. (f) *DAB2* expression was positively correlated with MS4A1 expression in DTC samples. (g) *DAB2* expression was positively correlated with KLRD1 expression in DTC samples. (h) *DAB2* expression was positively correlated with PDL1 expression in DTC samples. ^∗^*P* < 0.05, ^∗∗^*P* < 0.01, ^∗∗∗^*P* < 0.001, ^∗∗∗∗^*P* < 0.0001.

According to the result of KEGG analysis, *DAB2* may participate in the T cell receptor signaling pathway (Figure S3B). To further investigate the association between *DAB2* expression and immune microenvironment in DTC, the GSEA, ESTIMATE, and xCell analysis were performed. We found that some antitumor immunity related pathways were enriched in High *DAB2* group, such as T cell receptor signaling pathway, natural killer cell mediated cytotoxicity, antigen processing and presentation, and B cell receptor signaling pathway. (Figure 9(a)). Both the xCell and ESTIMATE analysis showed that High *DAB2* group had higher abundance of immune infiltration score than Low *DAB2* group (Figures 9(b) and 9(c)). The cell type infiltration abundance analysis showed that High *DAB2* group had higher infiltration abundance of CD4+memory/naïve T cells, CD8+T cells, B cells, and DC cells than Low *DAB2* group (Figure 9(d)). Moreover, we found that the expression of *DAB2* was positively correlated with the expression of classical biomarker of immune cells in PTC samples, such as *CD3G* (for T cells), *MS4A1* (for B cells), *KLRD1* (for NK cells), and immune checkpoint *PDL1* (Figures 9(e)–9(h)). Based on the above results, we inferred that PTC patients with high expression of *DAB2* would be benefit from immune-based therapy.

## 4. Discussion

Although there have been some studies on the relationship between abnormal DNA methylation and prognosis of DTC patients [21]. No reliable model considering the DNA methylation-driven gene signature to predict recurrence and metastasis risk accurately was reported before. In our study, we constructed a recurrence risk model with methylation profiles of *SPTA1, APCS,* and *DAB2* by stepwise multivariable Cox regression with data from TCGA. *SPTA1*, which have been linked to hereditary elliptocytosis and hereditary spherocytosis [45], were reported as a possible tumor driver gene in prostate cancer [46] and papillary thyroid carcinoma [47]. *APCS* is a gene that codes serum amyloid P-component, which is one of the main acute-phase reactants and has reported to a biomarker for survival in nonsmall cell lung cancer after thoracic radiotherapy [48]. Infrequent promoter hypermethylation of *DAB2* have been found to play a critical role in tumorigenesis and progression according to previous studies [24, 26]. Of note, we developed and validated a nomogram combined the methylation-related model, age, and AJCC_T for predicting the recurrence probability of DTC, which has not been published before to the best of our knowledge. DCA indicated that our model showed more net benefit for 5-year RFS than age, AJCC_T, He et al.'s, and Zhang et al.'s model, and when we combined our model with clinical parameters, DTC patients would gain the greatest benefit for 1-year, 3-year, and 5-year RFS.

Analysis of the 3 genes in the model at the transcriptional level showed that *DAB2* is a methylation-driven gene in DTC. The mRNA expression of *DAB2* was downregulated significantly in DTC compared to normal thyroid tissue. However, *SPTA1* and *APCS* both in DTC and normal thyroid tissues were in a state of hypermethylation and low expressed. Therefore, the function of *DAB2* was selected to be further explored in the DTC progression. Numerous literatures reported that *DAB2* involved in the migration, invasion, and proliferation of tumors [49–52]. However, the roles of *DAB2* in DTC have rarely been explored. In our study, we found that *DAB2* expression was lower in DTC tissues than that in normal thyroid tissues at the protein level, which was validated by tissue microarray staining for *DAB2* in specimens collected from Affiliated Hospital of Jining Medical University. Moreover, we found that overexpression of *DAB2* could inhibit proliferation and migration of BAPCP cells in vitro experiments. Enrichment of pathways using data from TCGA suggested that *DAB2* was relevant to some pathways that played various essential roles in the tumor growth and aggressive phenotypes such as NF-kappa B signaling pathway and PI3K-Akt signaling pathway. Activation of NF-kappa B is reported in DTC and correlates with tumor growth, drug-induced apoptosis [53, 54], and aggressiveness of DTC [55]. The activation of PI3K-Akt signaling pathway 395 also involves in the initiation and progression of thyroid cancer [56, 57]. Interestingly, we firstly focused on the relationship between *DAB2* expression and immune microenvironment in DTC. Pervious study reported that, loss of *DAB2* expression induced immune tolerance via accumulation of TGF-*β* in breast cancer [58]. In our study, we found that expression of *DAB2* was correlated with immunoscore in DTC samples and may be a biomarker for predicting the response to immune therapy in patients with DTC.

Although we successfully developed and validated a nomogram that may predict the recurrence of DTC patients, there were still some limitations in this study. Firstly, our external validating cohorts only included 115 DTC patients that were not completely sufficient, moreover, due to the insufficient clinical characteristics in the current study cohorts, there is a need to build a better prognostic nomogram from more centers with complete clinical information and sequencing data. Secondly, further experimental verification on molecular mechanism of *DAB2* in DTC is required to consolidate our results.

## 5. Conclusions

We successfully constructed and validated a nomogram based on methylation profiles of 3 genes, age, and AJCC_T for predicting recurrence probability of DTC patients. Besides, we found that promoter hypermethylation and loss expression of *DAB2* may be a biomarker of unfavorable prognosis and poor response to immune therapy in DTC.

## Data Availability

The main results of this study are based on public data from TCGA (https://portal.gdc.cancer.gov/) and GEO (https://www.ncbi.nlm.nih.gov/geo/).The clinical information and IHC data for human specimens are shown in Table S4.

## Supplementary Materials

Supplementary Materials Figure S1. Removed batch effect between GSE51090 and GSE97466. Figure S2. Original blot and images. Figure S3. Enrichment of Kyoto Encyclopedia of Genes and Genomes (KEGG) pathways related to *DAB2*. A Identification of differential expression genes (DEGs) between DTC patients in High *DAB2* group and Low *DAB2* group. B. Bubble plot shows the top 20 significant pathways enriched in KEGG analysis using DEGs as input. KEGG, Kyoto Encyclopedia of Genes and Genomes (KEGG); DEGs, differential expression genes. Table S1. The clinical information of TCGA-THCA cohorts. RFS, recurrence free survival; OS, overall survival. Table S2. The clinical information of GSE51090 cohorts. RFS, recurrence free survival. Table S3. The clinical information of GSE97466 cohorts. RFS, recurrence free survival. Table S4. The clinical information and IHC data for human specimens IHC, Immunohistochemistry.
