# Supplementary material for: Identification and Validation of a Prognostic Signature Based on Methylation Profiles and Methylation-Driven Gene DAB2 as a Prognostic Biomarker in Differentiated Thyroid Carcinoma
Source: Dis Markers. 2022 Sep 17;2022:1686316. doi: 10.1155/2022/1686316 (PMC10202610; doi:10.1155/2022/1686316)

**Identification and validation of a prognostic signature based on methylation profiles and methylation-driven gene DAB2 as a prognostic biomarker in differentiated thyroid carcinoma**

Gaoda Ju[1,2,3,4#], Lingling Zhang[4#], Wenting Guo[2,3#], Hao Wang[5], Xin Zhang[2,3], Zhuanzhuan Mu[2,3], Yuqing Sun[2,3], Di Sun[2,3], Han Diao[6], Sen Miao[6], Yiran Chen[1], Tao Xing[1], Jun Liang[1,4], Yansong Lin[2,3*]

Figure 8A Original blot and images

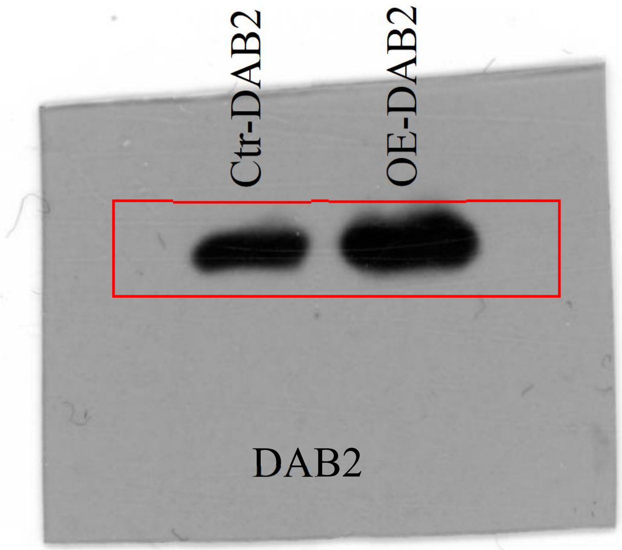


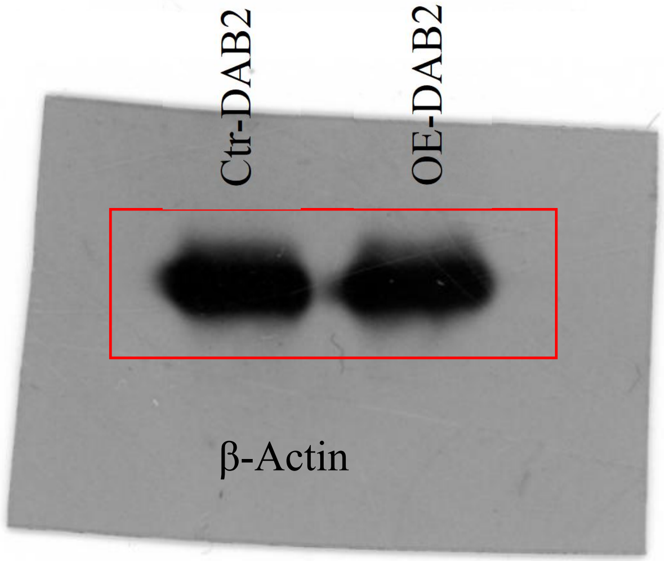

Supplement: Supplementary Materials — Figure S1. Removed batch effect between GSE51090 and GSE97466. Figure S2. Original blot and images. Figure S3. Enrichment of Kyoto Encyclopedia of Genes and Genomes (KEGG) pathways related to DAB2. A Identification of differential expression genes (DEGs) between DTC patients in High DAB2 group and Low DAB2 group. B. Bubble plot shows the top 20 significant pathways enriched in KEGG analysis using DEGs as input. KEGG, Kyoto Encyclopedia of Genes and Genomes (KEGG); DEGs, differential expression genes. Table S1. The clinical information of TCGA-THCA cohorts. RFS, recurrence free survival; OS, overall survival. Table S2. The clinical information of GSE51090 cohorts. RFS, recurrence free survival. Table S3. The clinical information of GSE97466 cohorts. RFS, recurrence free survival. Table S4. The clinical information and IHC data for human specimens IHC, Immunohistochemistry. [file 1686316.f1.zip › Figure S2 (1).pdf]
